# A facilitated diffusion model constrained by the probability isotherm: a pedagogical exercise in intuitive non-equilibrium thermodynamics

**DOI:** 10.1098/rsos.170429

**Published:** 2017-06-14

**Authors:** Brian Chapman

**Affiliations:** School of Applied and Biomedical Science, Faculty of Science and Technology, Federation University Australia, Northways Road, Churchill, Victoria 3842, Australia

**Keywords:** facilitated diffusion, probability isotherm, intuitive non-equilibrium thermodynamics, force–flux relations, saturation kinetics, Michaelis–Menten kinetics

## Abstract

This paper seeks to develop a more thermodynamically sound pedagogy for students of biological transport than is currently available from either of the competing schools of linear non-equilibrium thermodynamics (LNET) or Michaelis–Menten kinetics (MMK). To this end, a minimal model of facilitated diffusion was constructed comprising four reversible steps: *cis-*substrate binding, *cis*→*trans* bound enzyme shuttling, *trans*-substrate dissociation and *trans*→*cis* free enzyme shuttling. All model parameters were subject to the second law constraint of the probability isotherm, which determined the unidirectional and net rates for each step and for the overall reaction through the law of mass action. Rapid equilibration scenarios require sensitive ‘tuning’ of the thermodynamic binding parameters to the equilibrium substrate concentration. All non-equilibrium scenarios show sigmoidal force–flux relations, with only a minority of cases having their *quasi*-linear portions close to equilibrium. Few cases fulfil the expectations of MMK relating reaction rates to enzyme saturation. This new approach illuminates and extends the concept of rate-limiting steps by focusing on the free energy dissipation associated with each reaction step and thereby deducing its respective relative chemical impedance. The crucial importance of an enzyme's being thermodynamically ‘tuned’ to its particular task, dependent on the *cis-* and *trans-*substrate concentrations with which it deals, is consistent with the occurrence of numerous isoforms for enzymes that transport a given substrate in physiologically different circumstances. This approach to kinetic modelling, being aligned with neither MMK nor LNET, is best described as intuitive non-equilibrium thermodynamics, and is recommended as a useful adjunct to the design and interpretation of experiments in biotransport.

## 1. Introduction

*Aim.* The purpose of this paper is to remedy pedagogical error in matters pertaining to the thermodynamics and kinetics of biological transport phenomena. This is done by using a minimal model of facilitated diffusion, based on the mass action law and constrained by the probability isotherm, to provide straightforward thermodynamic and kinetic insights that elude the competing approaches of Michaelis–Menten kinetics (MMK) and linear non-equilibrium thermodynamics (LNET). This new approach is aptly named intuitive non-equilibrium thermodynamics (INET) for what, it is hoped, will become obvious reasons.

It is a persistent and dreary oral tradition in the folklore of chemical energetics pedagogy that ‘thermodynamics has nothing to say about reaction rates except at equilibrium’. This quite erroneous myth persists despite occasional attempts to dispel it by establishing consistency between non-equilibrium kinetics and thermodynamics. A significant attempt was published by Boudart in the mid-1970s for the case of complex unbranched chemical reactions [1] and was extended a decade later by Wagg to include all chemical, osmotic and chemiosmotic reactions, however complex, whether branched or unbranched [2]. This line of enquiry was pursued further by Wagg and co-workers in the 1990s for theoretical analysis of membrane transport [3–5], although an important limitation affecting experimental application of such theory is the inability of radioisotopic fluxes to distinguish between the various entry and exit points of ions involved in branched transport mechanisms [6].

Meanwhile, another attempt to relate thermodynamics to non-equilibrium reaction kinetics was initiated by Porter in 1983 [7] who derived from the van't Hoff isotherm a curiously expressed formula relating a reaction's molar free energy change (Δ*µ*) to the net rate (*j*) and unidirectional forward rate (*J*) of the reaction, thus

1.1 $$\Delta \mu =\text{RT}\ln\left[1-\left(\frac{j}{J}\right)\right],$$

where *R* and *T* have their usual meanings. While we shall have no further use for this formulation—it will be seen that it is identical to the rate isotherm defined below as equation (2.3), though much less intuitively expressed—Porter's derivation was important because it set down the required condition for assumption of Boltzmann equilibrium in liquid-phase media that the reaction times be longer than 10^−11^ s. This condition, permitting application of the mass action law using equilibrium-determined rate constants into the non-equilibrium domain, is amply satisfied for processes occurring under biological conditions of temperature and pressure, where the second-order diffusion-limited binding constants are orders of magnitude slower than 10^11^ s^−1^, and first-order protein conformational changes are orders of magnitude slower again (see table 1 and associated text).

**Table 1.** Thermodynamic and kinetic parameters for a minimal model of facilitated diffusion.

		rate coefficients		
step	reaction	forward	backward	$\Delta G_{i=1,2,3,4}^{o}$
1	${\text{A}}_{1}+{\text{E}}_{1}\overset{k_{1}}{\underset{k_{-1}}{\;⇌}}{\text{E}}_{1}\text{A}$	*_k_*__1_ = 10_^7^_ M_^−1^ _s_^−1^	$k_{-1}=k_{1}/\exp(-\Delta G_{1}^{o}/RT)\;{\text{s}}^{-1}$	$\Delta G_{1}^{o}=-RT\ln(k_{1}\text{/}k_{-1})$
2	${\text{E}}_{1}\text{A}\overset{k_{2}}{\underset{k_{-2}}{\;⇌}}{\text{E}}_{2}\text{A}$	$k_{2}\leq 10^{4}\;{\text{s}}^{-1}$	$k_{-2}\leq 10^{4}\;{\text{s}}^{-1}$	ΔG2o=−RTln ⁡k2k−2
		when $\Delta G_{2}^{o}\leq 0,$ then		
		$k_{2}=10^{4}\;{\text{s}}^{-1}\;$ and $\;k_{-2}=k_{2}\text{/}{\text{e}}^{-\Delta G_{2}^{o}\text{/}RT}\;{\text{s}}^{-1}$		
		when $\Delta G_{2}^{o}>0,$ then		
		$k_{2}=k_{-2}{\text{e}}^{-\Delta G_{2}^{o}\text{/}RT}\;{\text{s}}^{-1}\;$ and $\;k_{-2}=10^{4}\;{\text{s}}^{-1}$		
3	${\text{E}}_{2}\text{A}\overset{k_{3}}{\underset{k_{-3}}{\;⇌}}{\text{A}}_{2}+{\text{E}}_{2}$	$k_{3}=k_{-3}\cdot \exp(-\Delta G_{3}^{o}\text{/}RT)\;{\text{s}}^{-1}$	$k_{-3}=10^{7}\;{\text{M}}^{-1}\;{\text{s}}^{-1}$	$\Delta G_{3}^{o}=-RT\ln(k_{3}\text{/}k_{-3})$
4	${\text{E}}_{2}\overset{k_{4}}{\underset{k_{-4}}{\;⇌}}{\text{E}}_{1}$	$k_{4}\leq 10^{4}\;{\text{s}}^{-1},$	$k_{-4}\leq 10^{4}\;{\text{s}}^{-1}$	$\Delta G_{4}^{o}=-RT\ln(k_{4}\text{/}k_{-4})$
		when $\Delta G_{4}^{o}\leq 0,$ then		
		$k_{4}=10^{4}\;{\text{s}}^{-1}\;$ and $\;k_{-4}=k_{4}\text{/}{\text{e}}^{-\Delta G_{4}^{o}\text{/}RT}\;{\text{s}}^{-1}$		
		when $\Delta G_{4}^{o}>0,$ then		
		$k_{4}=k_{-4}{\text{e}}^{-\Delta G_{4}^{o}\text{/}RT}\;{\text{s}}^{-1}\;$ and $\;k_{-4}=10^{4}\;{\text{s}}^{-1}$		
overall	${\text{A}}_{1}⇌{\text{A}}_{2}$	—	—	$\begin{array}{l} \Delta G_{\mathrm{overall}}^{o}\\ =\sum_{i=1}^{i=4}\Delta G_{i}^{o}\\ =0 \end{array}$

Six years ago, three strands of pedagogical error in bioenergetics that have persisted since the 1970s were identified [8] and tracked historically [9] in papers that (i) identified the probability isotherm as an intuitive non-equilibrium thermodynamic framework for biochemical kinetics and (ii) stressed the importance of distinguishing between the frequently confounded concepts of *entropy creation* and *entropy exchange*. Last year, the probability isotherm was applied to offer an alternative interpretation of experimental data obtained on the F_o_F_1_-ATPase [10], echoing a much earlier instance of its application in 1980 to re-interpreting the kinetics of *n*, *m* and *h* in the Hodgkin–Huxley equations pertaining to electrical excitation in nerves [11]. While such application of the probability isotherm (though not under that name) is now commonplace in biological modelling of voltage gating mechanisms, it is rarer than it should be in relation to modelling of osmotic and chemiosmotic systems in biomembrane transport. It is perhaps a reflection of this rarity that the authors of the more recent application of the probability isotherm to the F_o_F_1_-ATPase [10] were pleasantly surprised by being prevailed upon in the review process to provide a pedagogical appendix on the distinction between entropy creation and entropy exchange and its relation to thermodynamic efficiency.

Thus, there would seem to be a need to develop a more robust pedagogy for scientists at the cutting edge of research into biological transport. The present paper uses a numerical simulation of a minimal model of facilitated diffusion to illustrate how such a pedagogy might be developed.

## 2. Technical definition of the probability isotherm and the rate isotherm

The probability isotherm affords a thermodynamic definition of a *probability ratio* for forward and backward reaction, *p*_f_/*p*_b_, thus

2.1 $$\Delta G_{\mathrm{Diss}}=RT\ln\left(\frac{p_{f}}{p_{b}}\right),$$

where Δ*G*_Diss_ is the molar free energy dissipation of the reaction. It should be noted that the unidirectional probabilities are individually undefined; only their *ratio* is defined according to the second law of thermodynamics as expressed in equation (2.1). As shown elsewhere [8], the probability ratio given in equation (2.1) is the product of an *intrinsic* probability ratio (equal to the equilibrium constant, *K*_eq_) determined by the *nature* of the reactants and products, and an *extrinsic* probability ratio determined by the *composition* (concentrations of reactants and products). As with all thermodynamic relationships, equation (2.1) is totally aloof from mechanism, so much so that it remains true regardless of whether or not any reaction mechanism actually exists.

For example, the oxidation of glucose according to

2.2 $${\text{C}}_{6}{\text{H}}_{12}{\text{O}}_{6}+6{\text{O}}_{2}\to \text{6C}{\text{O}}_{2}+6{\text{H}}_{2}\text{O},$$

yields 2.87 MJ per mole of glucose under standard conditions; for *in vivo* conditions in respiring animals and plants, the value is somewhat less at around 2.85 MJ mol^−1^. The probability isotherm determines a probability ratio for forward to reverse directions of reaction (2.2) at 37 K *in vivo* equal to *e*^1108^, a number with a magnitude beyond the RAM capacity of today's personal computers; its value might be made more accessible to current computing technology and human appreciation by representing it numerically as the alternative expression ${\left(1.33\times 10^{48}\right)}^{10}$.

However, this does not mean that glucose (or a glucose-containing organism) forms an explosive mix with air. Fortunately, there is no known mechanism for the direct oxidation of glucose by molecular oxygen; while glucose may be heated in air, only residual carbon is oxidized following thermal dehydration. Nonetheless, the probability ratio for glucose oxidation is thermodynamically defined according to equation (2.1) and sets a benchmark into which all thermodynamically determined probability ratios for the known partial reactions of intermediary metabolism of glucose must ‘fit’. While this ‘fitting’ requirement often goes under the quaint name of ‘detailed balance’, the present author prefers the ‘second law’ designation of what is essentially a thermodynamic requirement.

In cases where there are known mechanisms for reaction, the molar free energy dissipation also determines a rate isotherm giving a *rate ratio* for forward and backward reaction, *r*_f_/*r*_b_, identical to the probability ratio determined by the probability isotherm, thus

2.3 $$\Delta G_{\mathrm{Diss}}=RT\ln\left(\frac{r_{f}}{r_{b}}\right).$$

In this case, the unidirectional rates, unlike the unidirectional probabilities in equation (2.1), are actually defined and are quantitatively determined according to whatever level of catalysis might be present for any given composition of reactants and products. However, while the level of catalysis may influence the absolute values of the unidirectional rates, it cannot possibly influence their *ratio* which is always and everywhere determined thermodynamically. Even though equation (2.3) is a kinetic expression of the more fundamental equation (2.1), its thermodynamic message is that the level of catalysis cannot influence the rate ratio any more than it can influence the position of thermodynamic equilibrium. In this sense, therefore, thermodynamics has a great deal to say about non-equilibrium kinetics, and equation (2.3) affords a quantitative constraint that can be brought into play to enhance the design and interpretation of studies of membrane transport in which the thermodynamic boundary conditions are under experimental control.

Equations (2.1) and (2.3) apply at all times to all chemical, osmotic and chemiosmotic reactions; in the case of complex reactions, they apply equally to overall reactions and to individual reaction steps. As mentioned above, the rate isotherm expressed in equation (2.3) is equivalent to Porter's formulation shown in equation (1.1), where Porter's Δ*μ* is equal to the rate isotherm's –Δ*G*_Diss_ and his expression in parenthesis is equal to *r*_b_/*r*_f_, i.e. the reciprocal of the corresponding expression in the rate isotherm. However, the rate isotherm is more intuitively expressed in that it relates an increase in molar free energy dissipation directly to an increasing ratio of forward to backward rates of reaction.

## 3. A minimal model of facilitated diffusion (uniport)

Consider the osmotic reaction

$${\text{A}}_{1}⇌{\text{A}}_{2},$$

whereby a substrate, A, is translocated across a membrane separating two aqueous compartments, 1 (*cis-*) and 2 (*trans-*), respectively. The translocation mechanism involves a transmembrane uniporter enzyme, E, having two conformational states, E_1_ and E_2_, respectively, and catalysing the reaction according to the following mechanism comprising four elementary steps:

$$\begin{array}{rlrl} {\text{A}}_{1}+{\text{E}}_{1} & \overset{k_{1}}{\underset{k_{-1}}{\;⇌}}\text{A}{\text{E}}_{1}\; & & \text{Step}\;1,\\ \text{A}{\text{E}}_{1} & \overset{k_{2}}{\underset{k_{-2}}{\;⇌}}\text{A}{\text{E}}_{2}\; & & \text{Step}\;2,\\ \text{A}{\text{E}}_{2} & \overset{k_{3}}{\underset{k_{-3}}{\;⇌}}{\text{A}}_{2}+{\text{E}}_{2}\; & & \text{Step}\;3\\ \text{and}\;\;{\text{E}}_{2} & \overset{k_{4}}{\underset{k_{-4}}{\;⇌}}{\text{E}}_{1}\; & & \text{Step}\;4, \end{array}$$

where *k_i_* and *k*_−*i*_ are the respective forward and backward rate coefficients of the *i*th elementary step.

For a passive process of facilitated diffusion, the standard molar free energy change of the overall reaction, $\Delta G_{{\text{A}}_{1}\to {\text{A}}_{2}}^{o}$, is zero, i.e. there is no intrinsically preferred location for the substrate A. This means that there is no asymmetry of intrinsic probability for the movement of A in either direction; in other words, the intrinsic probability ratio for movement of A in either direction is unity and the actual probability ratio will be given only by the concentration ratio for A in the two compartments.

In order to work with a model that can be related to the actual performance of real transport processes reported in the biomedical literature, we shall aim to ‘design’ a uniporter enzyme that can achieve a membrane transport density of the order of 1 pmol cm^−2^ s^−1^ with a maximum molecular turnover number of 1000 s^−1^ and requiring an enzyme site density of around 1 fmol cm^−2^. In accordance with the simpler enzymatic function of a uniporter, this turnover number is roughly ten times that reported for the more complex chemiosmotic Na^+^,K^+^-ATPase [12] while being comparable to that found for the glucose transporter 1 (GLUT1) that mediates facilitated diffusion [13].

### 3.1. Assumptions about the rate coefficients

#### 3.1.1. Enzyme substrate binding

We assume that the second-order association constants *k*_1_ and *k*_−3_ are both 10^7^ M^−1^ s^−1^, i.e. a ‘middle-order’ value in the commonly encountered range of 10^6^–10^8^ M^−1^ s^−1^ for diffusion-limited ligand–receptor binding [14]. This means that the first-order dissociation constants *k*_−1_ and *k*_3_ will be thermodynamically determined by the standard free energies, $\Delta G_{1}^{o}$ and $\Delta G_{3}^{o}$, chosen for steps 1 and 3, respectively, as shown in table 1.

#### 3.1.2. Conformational changes (translocation)

We assume that the first-order turnover numbers *k*_2_, *k*_−2_, *k*_4_ and *k*_−4_ are limited to being no greater than 10^4^ s^−1^ (corresponding to the upper limit of reported protein conformational turnover numbers). When exploring the effects of variation in the standard free energies, $\Delta G_{2}^{o}$ and $\Delta G_{4}^{o}$, chosen for steps 2 and 4, respectively, it is therefore necessary to impose the logical controls on the optimized rate coefficients for these steps shown in table 1.

Table 1 thus shows the kinetic and thermodynamic relationships of the parameters for each of the reaction steps. It may be seen that, within the stated constraints on the upper limit for the rate coefficients of either association or translocation, specification of a numerical value for the standard free energy, $\Delta G_{i}^{o}$, of the *i*th step will determine the absolute values of the forward and reverse rate coefficients, *k_i_* and *k_−i_*, for that step.

## 4. Simulation methods

### 4.1. Steady-state equations

The following four equations for the rates of change in concentration of the respective four forms of the enzyme apply in the steady state:

$$\begin{array}{rl} \frac{\text{d}[{\text{E}}_{1}]}{\text{d}t} & =-k_{1}\cdot [{\text{A]}}_{1}\cdot [{\text{E}}_{1}]+k_{-1}\cdot [{\text{E}}_{1}\text{A]}+k_{4}\cdot [{\text{E}}_{2}]-k_{-4}\cdot [{\text{E}}_{1}]=0,\\ \frac{\text{d}[{\text{E}}_{1}\text{A]}}{\text{d}t} & =k_{1}\cdot [{\text{A]}}_{1}\cdot [{\text{E}}_{1}]-k_{-1}\cdot [{\text{E}}_{1}\text{A]}-k_{2}\cdot [{\text{E}}_{1}\text{A]}+k_{-2}\cdot [{\text{E}}_{2}\text{A]}=0,\\ \frac{\text{d}[{\text{E}}_{2}\text{A]}}{\text{d}t} & =k_{2}\cdot [{\text{E}}_{1}\text{A]}-k_{-2}\cdot [{\text{E}}_{2}\text{A]}-k_{3}\cdot [{\text{E}}_{2}\text{A]}+k_{-3}\cdot [{\text{A]}}_{2}\cdot [{\text{E}}_{2}]=0\\ \text{and}\;\frac{\text{d}[{\text{E}}_{2}]}{\text{d}t} & =k_{3}\cdot [{\text{E}}_{2}\text{A]}-k_{-3}\cdot [{\text{E}}_{2}]\cdot [{\text{A]}}_{2}-k_{4}\cdot [{\text{E}}_{2}]+k_{-4}\cdot [{\text{E}}_{1}]=0. \end{array}$$

Of these four equations in four unknowns, only three are independent. The fourth independent equation required for steady-state solution is the conservation equation for the total concentration of all forms of the enzyme, thus

4.1 $$[{\text{E}}_{1}]+[{\text{E}}_{1}\text{A}]+[{\text{E}}_{2}\text{A}]+[{\text{E}}_{2}]=[\text{total enzyme}].$$

### 4.2. Mathematical methods

These steady-state equations were solved numerically using the algorithms built into Microsoft Excel, the resulting concentrations of the four forms of the enzyme then being used to calculate the unidirectional rates of each of the four reaction steps as given in table 2. The formulae for calculating the overall unidirectional rates were derived according the method given by Boudart [1], assuming a value of unity for the stoichiometric number (Boudart's *χ_i_*) of each of the reaction steps 1–4, as given in the ‘overall’ row of table 2. The concentration of total enzyme was set at 1 fmol cm^−2^ of membrane between compartments 1 and 2, comparable to the density of Na^+^,K^+^-ATPase in plasmalemmal membranes reported in the literature [15]. The Excel spreadsheet (INET.xlsx) used for the calculations reported in this paper is supplied as the electronic supplementary material .

**Table 2.** Steady-state rates and free energy dissipation for a minimal model of facilitated diffusion. The free energy dissipation at each step is given by the respective rate isotherm, while that for the overall reaction is given by the overall probability isotherm, the thermodynamic *alter ego* of the van't Hoff isotherm.

		rates		
reaction	step	forward	backward	Δ*G*_diss_ (kJ mol^−1^)
1	${\text{A}}_{1}+{\text{E}}_{1}\overset{k_{1}}{\underset{k_{-1}}{\;⇌}}{\text{E}}_{1}\text{A}$	$r_{1}=k_{1}\cdot [{\text{A]}}_{1}\cdot [{\text{E}}_{1}]$	$r_{-1}=k_{-1}\cdot [{\text{E}}_{1}\text{A}]$	$RT\ln(r_{1}\text{/}r_{-1})$
2	${\text{E}}_{1}\text{A}\overset{k_{2}}{\underset{k_{-2}}{\;⇌}}{\text{E}}_{2}\text{A}$	$r_{2}=k_{2}\cdot [{\text{E}}_{1}\text{A}]$	$r_{-2}=k_{-2}\cdot [{\text{E}}_{2}\text{A}]$	$RT\ln(r_{2}\text{/}r_{-2})$
3	${\text{E}}_{2}\text{A}\overset{k_{3}}{\underset{k_{-3}}{\;⇌}}{\text{A}}_{2}+{\text{E}}_{2}$	$r_{3}=k_{3}\cdot [{\text{E}}_{2}\text{A}]$	$r_{-3}=k_{-3}\cdot [{\text{A]}}_{2}\cdot [{\text{E}}_{2}]$	$RT\ln(r_{3}\text{/}r_{-3})$
4	${\text{E}}_{2}\overset{k_{4}}{\underset{k_{-4}}{\;⇌}}{\text{E}}_{1}$	$r_{4}=k_{4}\cdot [{\text{E}}_{2}]$	$r_{-4}=k_{-4}\cdot [{\text{E}}_{1}]$	$RT\ln(r_{4}\text{/}r_{-4})$
overall	${\text{A}}_{1}⇌{\text{A}}_{2}$	$\left[r_{1}r_{2}r_{3}r_{4}\text{/denominator}\right]$	$\left[r_{-1}r_{-2}r_{-3}r_{-4}\text{/denominator}\right]$	$RT\ln({\left[A\right]}_{1}\text{/}{\left[A\right]}_{2})$
	where, following Boudart [1], the $\text{denominator}=r_{2}r_{3}r_{4}+r_{-1}r_{3}r_{4}+r_{-1}r_{-2}r_{4}+r_{-1}r_{-2}r_{-3}$.			

### 4.3. Choosing and sweeping the independent variable(s); display of data

The studies described here examine the influence of (i) the affinity of the enzyme for the substrate, (ii) the absolute concentration of the substrate and (iii) the concentration gradient of the substrate. This was done by using ToolBook to set the respective boundary conditions and sweep the independent variable(s) by passing them as input variables to Excel via dynamic data exchange. The primary solutions of the four linear equations and the secondary calculations of rates, enzyme saturation and free energy dissipation were all performed within Excel, and selected results were extracted by ToolBook via dynamic data exchange and plotted graphically within ToolBook. ToolBook's graphical displays were saved as screen captures and converted to PDFs for printing.

Using ToolBook to pass parameters to Excel and to retrieve solutions therefrom through dynamic data exchange was much faster than performing the primary and secondary operations within ToolBook, provided that the open Excel file was kept minimized during any given simulation run. As recorded in the Acknowledgements, the accuracy of these unorthodox numerical methods was initially cross-checked using Matlab by colleagues at the University of Auckland. Once this accuracy was thus confirmed, all further virtual experimental scenarios were simulated using this ToolBook–Excel regime. The heuristic and pedagogical advantages of constructing the numerical simulation in Excel should become obvious on perusal of the INET.xlsx file provided.

## 5. Numerical simulation of model behaviour

### 5.1. Rapid equilibration across a barrier—effects of binding affinity

Rapid equilibration across biomembranes is not commonly found for transport of ions or metabolites, but it is common for water transport subserved by several different aquaporin proteins. Nonetheless, our heuristic purposes are well served by examining the factors that optimize the ability of a uniporter to facilitate rapid equilibration. The design specification for such a uniporter might reasonably be supposed to involve (i) perfect symmetry for the *cis-* and *trans-*binding affinities (i.e. $\Delta G_{1}^{o}=-\Delta G_{3}^{o}$) and (ii) no intrinsic preferred conformational state for either the bound or unbound forms of the enzyme (i.e. $\Delta G_{2}^{o}=\Delta G_{4}^{o}=0$).

We begin by sweeping the standard free energies of binding for steps 2 and 4 identically over the domain of –3*RT*ln(10) (high affinity) to +3*RT*ln(10) (low affinity) with unit substrate concentration (1 M) in both compartments. It is important to note that the thermodynamic constraint on the rate coefficients for the binding steps was realized by fixing the second-order diffusion-limited association constants *k*_1_ and *k*_−3_ at 10^7^ M^−1^ s^−1^ while allowing the enzyme-determined dissociation constants *k*_−1_ and *k*_3_ to take on various values constrained by the formulae given in table 1. This is equivalent to simulating the behaviour of a continuum of enzyme isoforms, each isoform having a different enzyme-specific dissociation constant.

**Figure 1.**
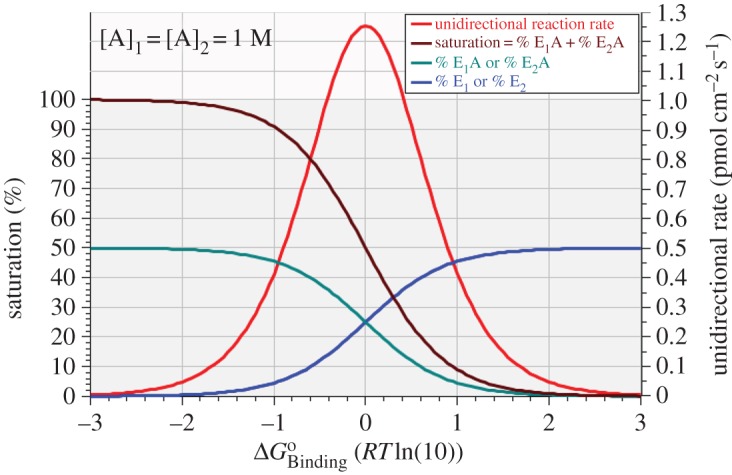
Enzyme saturation (left axis) and unidirectional reaction rate (right axis) versus standard free energy of binding for unit concentration (1 M) of substrate A in both compartments.

The plots displayed in figure 1 show that, with equal *cis-* and *trans*-concentrations of substrate, the percentages of the two bound forms of the enzyme superimpose at all levels of binding affinity, as do the percentages of the two free (unbound) forms. Because all the conditions are at equilibrium, the forward and backward rate curves also superimpose, while the net rate is zero throughout. In this specific case, where the substrate concentration is unity (1 M) in both compartments, the enzyme is exactly half saturated at zero standard free energy of binding, with each of the four enzyme forms being equally represented at 25%. Curiously, the maximum unidirectional rates, $R_{\max}^{+}=R_{\max}^{-}=1.2488\;\text{pmol}\;\text{c}{\text{m}}^{-2}\;{\text{s}}^{-1}$, are *not* achieved at exactly this point of 50% saturation with unit substrate concentration each side of the membrane, but at a saturation level of 49.9750%, with $\Delta G_{\mathrm{Binding}}^{o}$ slightly greater than zero, being equal to +4.3411 × 10^−4^*RT*ln(10) or +2.5776 J mol^−1^, such that *k*_−1_ = *k*_3_ = 1.0010 × 10^7^ s^−1^. This discrepancy between the affinity level corresponding to exactly 50% enzyme saturation and that corresponding to maximum unidirectional rates becomes progressively larger as the equilibrium substrate concentration is lowered and is owing to the fact that, while 50% saturation exactly follows the reciprocal changes in binding affinity and concentration, the denominator used for calculating the overall unidirectional rates (table 2) does not show such constancy in the face of reciprocal variation in substrate concentration and binding affinity.

**Figure 2.**
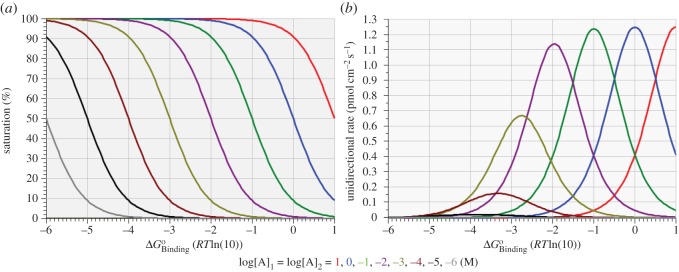
Variation of enzyme saturation (*a*) and unidirectional reaction rate (*b*) with standard free energy of binding for eight different values of equilibrium substrate concentration.

This effect is illustrated in figure 2 which shows the influence of binding affinity on equilibrium saturation levels (figure 2*a*) and unidirectional forward rates (figure 2*b*) at fixed levels of equilibrium substrate concentration ranging from 10^−6^ to 10 M. The conditions determining the maximum equilibrium unidirectional rate ($R_{\max}^{+}=R_{\max}^{-}$) at each of the eight stepped values of equilibrium substrate concentration are shown in table 3.

**Table 3.** Equilibrium substrate concentrations and enzyme saturation levels for maximum equilibrium unidirectional reaction rates ($R_{\max}^{+}=R_{\max}^{-}$) achieved with various binding affinities.

log[A]_1_ = log[A]_2_ (M)	$\Delta G_{\mathrm{Binding}}^{o}(RT\text{ln}(10))$	$R_{\max}^{+}=R_{\max}^{-}({\text{pmol cm}}^{-2}\;{\text{s}}^{-1})$	saturation (%)
1	1.000044113	1.249875016	49.997460652
0	0.000434106	1.248751560	49.975010855
−1	−0.995700528	1.237654095	49.752504512
−2	−1.960413065	1.138721247	47.722614266
−3	−2.761440632	0.669872981	36.602607666
−4	−3.338892551	0.160435608	17.912952906
−5	−3.848412668	0.021705638	6.588875043
−6	−4.349407913	0.002390669	2.186781851

Note that the maximum unidirectional rate achieved by different binding affinities is relatively constant over a substrate concentration domain of 10 M to 10 mM and is achieved at saturation levels of around 50% or slightly less; however, over the substrate concentration domain of 1 mM to 1 µM, $R_{\max}^{+}$ becomes greatly reduced and is achieved only at progressively lower saturation levels. As shown in table 3, the optimal binding affinity at each substrate concentration is always less than might be expected from a logarithmic relation with the substrate concentration—slightly less at high concentrations (less negative standard free energies) and progressively much less at low concentrations. On the other hand, the saturation plots of figure 2 confirm the straightforward expectation that the substrate concentration at which 50% saturation is achieved always follows exactly the relative change in binding affinity, i.e. a 10-fold change in 50% saturating concentration of substrate for every *RT*ln(10) unit of change in $\Delta G_{\mathrm{Binding}}^{o}$.

To assist with a perspective on the simulations recorded in figures 1 and 2, the continuous variation of $\Delta G_{\mathrm{Binding}}^{o}$ along the abscissae of these two figures implies a continuum of virtual isoforms of a particular species of uniporter, with each virtual isoform exposed to either 1 M (figure 1) or eight different (figure 2) equilibrium substrate concentrations on each side of the membrane. In particular, none of the curves shown in figures 1 and 2 represents the kinetic behaviour of any particular virtual isoform of a uniporter. In the next section, we show results that will indeed pertain to single virtual isoforms of a uniporter exposed to different equilibrium substrate concentrations.

### 5.2. Rapid equilibration across a barrier—effects of substrate concentration

**Figure 3.**
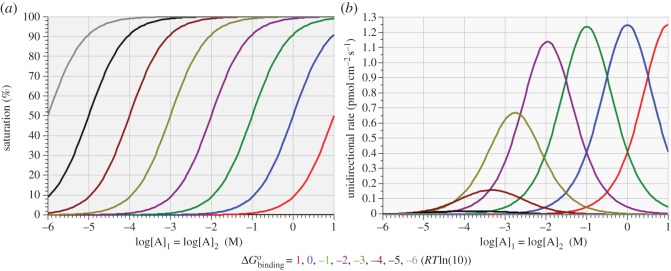
Variation of enzyme saturation (*a*) and unidirectional reaction rate (*b*) with equilibrium substrate concentration for eight different values of standard free energy of binding.

The results shown in figure 2 and table 3 confirm that, for any given equilibrium substrate concentration, there exists a unique binding affinity at which the equilibrium unidirectional rates are maximal. The complementary expectation—that, for any given binding affinity, there exists a unique equilibrium substrate concentration at which the equilibrium unidirectional rates are maximal—is confirmed by the results displayed in figure 3 and table 4, showing the effect of sweeping the equilibrium substrate concentrations over the domain of 1 µM to 10 M with the free energy of binding fixed at exact integer multiples, *m*, of *RT*ln(10), where –6 ≤ *m* ≤ 1. The curves shown in figure 3 thus pertain to eight different virtual isoforms of the uniporter.

**Table 4.** Equilibrium substrate concentrations and enzyme saturation levels for maximum unidirectional reaction rates achieved with various binding affinities.

$\Delta G_{\mathrm{Binding}}^{o}(RT\text{ln}(10))$	[A]_1_ = [A]_2_ (M)	log[A]_1_ = log[A]_2_ (M)	$R_{\max}^{+}=R_{\max}^{-}({\text{pmol cm}}^{-2}{\text{s}}^{-1})$	saturation (%)
1	10.001015515	1.000044101	1.249875016	50.002538661
0	1.001000294	0.000434205	1.248751560	50.024994849
−1	0.100994898	−0.995700567	1.237654095	50.247493345
−2	0.010954431	−1.960410195	1.138721247	52.277396600
−3	0.001732047	−2.761440452	0.669872981	63.397408613
−4	0.000458256	−3.338892313	0.160435607	82.087071165
−5	0.000141771	−3.848414128	0.021705638	93.411125972
−6	0.000044728	−4.349425375	0.002390668	97.813156053

A particularly interesting observation arising from these ‘complementary’ experiments is that, at each *RT*ln(10) unit of variation in the binding affinity or 10-fold variation of the substrate concentration, the equilibrium value of *R*_max_ is identical (within the limits of numerical precision) even though the enzyme saturation level at which it is achieved is different, with the differences in saturation becoming very large at low substrate concentrations. Moreover, there is a clear ‘symmetry’ between the optimal substrate concentration and the optimal binding affinity to achieve *R*_max_ at each decade of variation, as reflected by the essentially identical numerical value of the logarithm of optimal substrate concentration (table 4, column 3) and the number of *RT*ln(10) units of binding affinity (table 3, column 2). This ‘symmetry’ also extends to the deviation from 50% enzyme saturation for the *R*_max_ achieved at each decade of variation: the optimal saturation levels recorded for each decade in tables 3 and 4 deviate by identical amounts from 50% such that the average enzyme saturation recorded in the two tables for *R*_max_ is exactly 50% at every decade, regardless of how far the individual optimal saturation deviates from 50%.

### 5.3. Rapid equilibration across a barrier—disclosure of isokines

**Figure 4.**
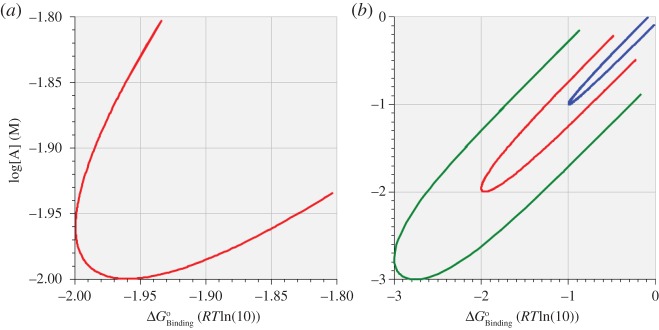
(*a*,*b*) Isokines for three different equilibrium unidirectional rates of 1.1387 pmol cm^−2^ s^−1^ (red curves), 1.2376 pmol cm^−2^ s^−1^ (blue curve) and 0.6699 pmol cm^−2^ s^−1^ (green curve) equal to the *R*_max_ values recorded in data rows 4,3 and 5, respectively, of tables 3 and 4. The enzyme saturation increases monotonically clockwise round each isokine and is exactly 50% at the point at which each curve intersects its axis of symmetry.

The symmetry observed between the sets of data presented in figures 2 and 3 and tables 3 and 4 suggests that there exist pathways in the concentration–affinity space upon which the equilibrium unidirectional rate remains constant; we shall call such pathways ‘isokines’. The isokine pertaining to data rows 4 of tables 3 and 4 (i.e. for log[A]_1_ = log[A]_2_ = –2(M) in table 3 and for $\Delta G_{\mathrm{Binding}}^{o}=-2RT\text{ln}(10)$ in table 4) is displayed in figure 4*a* over the domain −1.8,−1.8 to −2.0,−2.0 (figure 4*a*) and over the domain 0,0 to −3.0,−3.0 (figure 4*b*, red curve). Figure 4*b* also shows isokines pertaining to rows 3 (blue curve) and rows 5 (green curve) of tables 3 and 4. The data for figure 4*a* were obtained by stepping log[A]_1_ = log[A]_2_ over the required domain and, at each step, stepping $\Delta G_{\mathrm{Binding}}^{o}$ over the same numerical domain, and recording all values of concentration, affinity, saturation and equilibrium unidirectional rate (Rate) for which Rate had a value within a specified tolerance of the value of *R*_max_ = 1.1387 pmol cm^−2^ s^−1^ recorded in data rows 4 of tables 3 and 4. While the methods of generating the isokines in figure 4*a,b* were identical, the tolerance used for the curve in figure 4*a* was 10^−4^% while that used to generate the curves in figure 4*b* varied between 10^−4^% and 0.05% according to the required grid resolution.

The isokine shown in figure 4*a* has the appearance of a parabola tilted at 45° to the perpendicular, although it is clearly not parabolic (figure 4*b*, red curve). As one proceeds clockwise round the left-hand red isokine, the enzyme saturation increases monotonically from 42.52% to 57.48%. For the right-hand red isokine, the saturation increases monotonically clockwise from 35.25% to 64.75%. For each of the red isokines, the point corresponding to exactly 50.00% saturation occurs at the point of intersection of each curve with its axis of symmetry (the 45° line given by $\text{log}[\text{A}]=\Delta G_{\mathrm{Binding}}^{o}$). For the two red isokines, this point occurs when $\text{log[A] }=\Delta G_{\mathrm{Binding}}^{o}=-1.9899\;RT\text{ln}(10)$. However, this point does not correspond to *R*_max_ for the virtual isoform for which $\Delta G_{\mathrm{Binding}}^{o}=-1.9899\;RT\text{ln}(10)$; for that particular virtual isoform, *R*_max_ = 1.1410 pmol cm^−2^ s^−1^ when the equilibrium substrate concentration is 11.19 mM at 52.23% saturation.

Similar behaviour is seen for the blue and green isokines of figure 4*b*. For the blue isokine (Rate = 1.2377 pmol cm^−2^ s^−1^), as one proceeds clockwise the enzyme saturation increases monotonically from 45.41% to 54.59%, with exact 50% saturation occurring for a virtual isoform having $\Delta G_{\mathrm{Binding}}^{o}=-0.99\;RT\text{ln}(10)$. This virtual isoform has its own *R*_max_ = 1.2379 pmol cm^−2^ s^−1^ when the equilibrium substrate concentration is 103.32 mM at 50.24% saturation.

For the green isokine (Rate = 0.6699 pmol cm^−2^ s^−1^), the enzyme saturation increases monotonically clockwise from 16.00% to 84.00%, with exact 50% saturation occurring for a virtual isoform having $\Delta G_{\mathrm{Binding}}^{o}=-2.9375\;RT\text{ln}(10)$. This virtual isoform has its own *R*_max_ = 0.7105 pmol cm^−2^ s^−1^ when the equilibrium substrate concentration is 1.91 mM at 62.30% saturation.

The concept of theoretical isokines developed here demonstrates that different uniporter isoforms are capable of operating at similar absolute rates in the face of different substrate concentrations found in different environments. This kind of concept could be explored experimentally by comparing and contrasting different uniporter enzymes inserted in lipid vesicles, as is becoming increasingly possible through the evolving techniques for micro-measurement of fluxes and conformational transitions of enzymes.

It is worth noting that the concentration/affinity isokine and its associated continuum of variation of enzyme saturation are theoretical concepts of potentially significant experimental consequence that find no resonance in pedagogies deriving from LNET or MMK.

### 5.4. Rapid equilibration across a barrier—effects of translocation affinity

The data produced so far have all been obtained on the assumption that there is no intrinsic translocational preference between *cis-* and *trans-*conformations for either the bound or unbound forms of the enzyme (i.e. $\Delta G_{2}^{o}=\Delta G_{4}^{o}=0$). If such a preference is introduced, then, given the assumed symmetry of binding (i.e. $\Delta G_{1}^{o}=-\Delta G_{3}^{o}$), it must be based on similar ‘symmetry’, i.e. $\Delta G_{2}^{o}=-\Delta G_{4}^{o}$, in order to satisfy the second law requirement for the overall process that $\Delta G_{\mathrm{Overall}}^{o}=0$.

**Figure 5.**
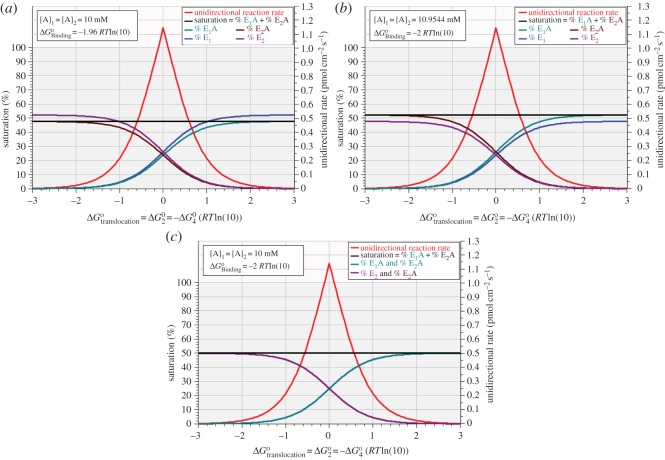
Enzyme saturation (left axis) and unidirectional reaction rate (right axis) versus standard free energy of translocation for three different conditions of substrate concentration and binding affinity. (*a*) [A]_1_ = [A]_2_ = 10 mM and $\Delta G_{\mathrm{Binding}}^{o}=\Delta G_{1}^{o}=-\Delta G_{3}^{o}=-1.9604\;RT\text{ln}(10)$; (*b*) [A]_1_ = [A]_2_ = 10.9544 mM and $\Delta G_{\mathrm{Binding}}^{o}=-2\;RT\text{ln(10) }$ and (*c*) [A]_1_ = [A]_2_ = 10 mM and $\Delta G_{\mathrm{Binding}}^{o}=-2\;RT\text{ln(10) }$.

Figure 5 shows the variation of equilibrium unidirectional rate, enzyme saturation and enzyme distribution when $\Delta G_{2}^{o}(=-\Delta G_{4}^{o})$ is swept over the domain –3 *RT*ln(10) to +3 *RT*ln(10) under three different sets of conditions. The data of figure 5*a* were obtained under the same conditions that yielded *R*_max_ = 1.1387 pmol cm^−2^ s^−1^ in figure 2 and row 4 of table 3, i.e. [A]_1_ = [A]_2_ = 10 mM and $\Delta G_{1}^{o}=-\Delta G_{3}^{o}=-1.9604\;RT\text{ln}(10)$. The sharp symmetrical decline in unidirectional rate with increasing translocational affinity in either direction is due entirely to the combined thermodynamic and kinetic constraint requiring an exponential decline in one or other of the rate coefficients for both steps 2 and 4 as the translocational affinity is made increasingly non-zero (table 1). As expected, negative values for $\Delta G_{2}^{o}$ result in a preponderance of E_2_ forms of the enzyme while positive values for $\Delta G_{2}^{o}$ result in a preponderance of E_1_ forms of the enzyme. These variations are reciprocal in that the level of enzyme saturation remains constant throughout, despite the large variation in unidirectional rate. Figure 5*b* shows the ‘complementary’ results obtained under conditions yielding the same *R*_max_ = 1.1387 pmol cm^−2^ s^−1^ in figure 3 and row 4 of table 4, i.e. [A]_1_ = [A]_2_ = 10.9544 mM and $\Delta G_{1}^{o}=-\Delta G_{3}^{o}=-2\;RT\text{ln}(10)$. Figure 5*c* shows the results obtained for the suboptimal intermediate condition in which [A]_1_ = [A]_2_ = 10 mM and $\Delta G_{1}^{o}=-\Delta G_{3}^{o}=-2\;RT\text{ln}(10)$ and *R*_max_ = 1.1364 pmol cm^−2^ s^−1^; under these conditions the two conformational states of the enzyme are exactly half distributed between the bound and free forms.

The ‘symmetry’ already noted in relation to the data of tables 3 and 4 and figure 4 is reinforced by the graphical data shown in figure 5*a*,*b* where there is exact numerical equality between figure 5*a* solutions for the percentages of the enzyme forms E_1_, E_1_A, E_2_A and E_2_ and figure 5*b* solutions for the percentages of E_1_A, E_1_, E_2_ and E_2_A, respectively.

Within the functional context of rapid equilibration between compartments, the results shown in figure 5 invite the teleological supposition that there is a significant disadvantage in a uniporter's having a preferred conformation; this is because the rate-limiting constraint inherent in protein conformational changes becomes amplified on one of the respective unidirectional rate coefficients as the standard free energy of conformational change departs from zero.

We now turn our attention to non-equilibrium simulations involving facilitated diffusion down a concentration gradient.

### 5.5. Force–flux relations

As noted earlier, it is a central expectation of LNET [16] that, in the absence of complicating factors, the force–flux relation of a process should be linear, provided that the process is occurring not too far from equilibrium (a proviso that is commonly very elastic in its application). While it is a trivial expectation that the force–flux relation of a saturable system such as a uniporter enzyme will be sigmoidal, with a quasi-linear inflection point occurring between the saturating asymptotes in either direction, it is not clear as to whether the inflection point will always be found at or near equilibrium. The next simulations address this question by examining the model's kinetic behaviour under non-equilibrium conditions in which log[A]_1_ is swept over the molar domain of −6 to +1 at eight fixed integer values of log[A]_2_ over the same domain for three different fixed values of binding affinity.

**Figure 6.**
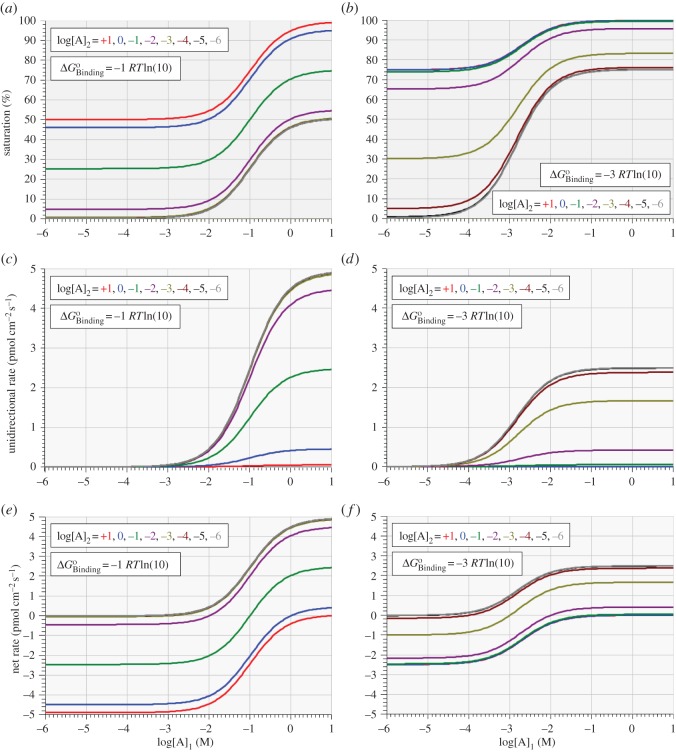
Variation of enzyme saturation (*a,b*), unidirectional forward rate (A_1→2_, *c,d*) and net reaction rate (A_1→2_ – A_2→1_, *e,f*) with *cis-*substrate concentration, [A]_1_, for different values of *trans-*substrate concentration, [A]_2_, with $\Delta G_{\mathrm{Binding}}^{o}$ equal to –1 (*a,c,e*) and –3 (*b,d,f*) times *RT*ln(10).

**Figure 7.**
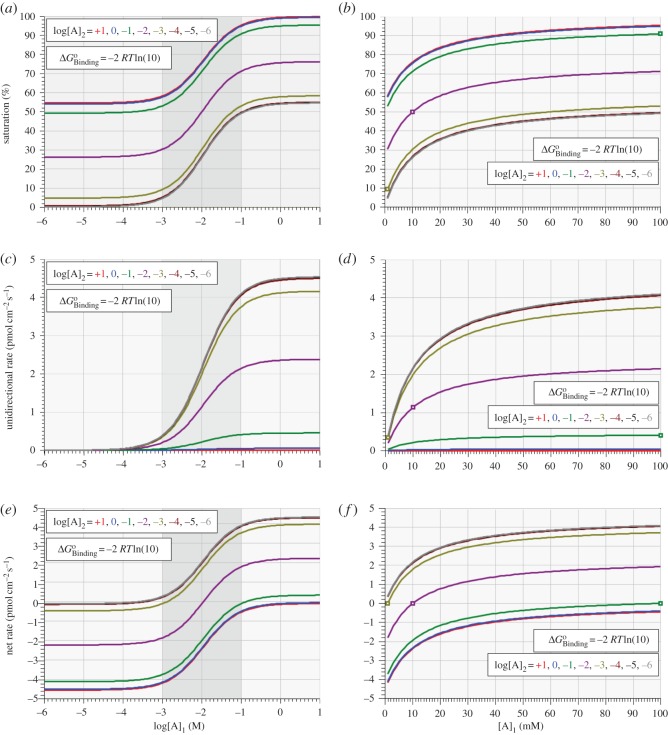
Variation of enzyme saturation (*a,b*), unidirectional forward rate (A_1→2_, *c,d*) and net reaction rate (A_1→2_ – A_2→1_, *e,f*) with *cis-*substrate concentration, [A]_1_, for different values of *trans-*substrate concentration, [A]_2_, with $\Delta G_{\mathrm{Binding}}^{o}=-2\;RT\text{ln( 10) }$. Panels (*b,d,f*) show linear plots expanded from the respective shaded domains of logarithmic plots of panels (*a,c,e*). Open squares in (*b,d,f*) mark the respective positions of thermodynamic equilibrium.

Figures 6*a*–*f* and 7*a,c,e* show plots of enzyme saturation (figures 6*a,b* and *7a*), unidirectional forward rate, A_1→2_ (figures 6*c,d* and *7c*) and net reaction rate (A_1→2_ – A_2→1_, figures 6*e,f* and *7e*) as functions of *cis-*substrate concentration, [A]_1_, for eight different fixed values of *trans-*substrate concentration, [A]_2_, with $\Delta G_{\mathrm{Binding}}^{o}$ fixed at –1 (figure 6*a,c,e*), –2 (figure 7*a,c,e*) and –3 (figure 6*b,d,f*) times *RT*ln(10).

Each graph of net rate versus log[A]_1_ shown in figures 6*e,f* and 7*e* are thermodynamic force–flux relations that all fulfil the intuitive expectation of being sigmoidal and showing saturating behaviour in both directions. These results thus confirm and extend the earlier demonstration by Chapman & Loiselle [10] that linear force–flux relations are not generally to be expected. Of all the non-equilibrium force–flux relations simulated here, only those obtained with $\Delta G_{\mathrm{Binding}}^{o}$ set symmetrically to *RT*ln(10) times the respective equilibrium value of log[A]_1_ = log[A]_2_ have their *quasi*-linear inflection points approximately centred at equilibrium; all other force–flux relations have their inflection points centred both away from equilibrium and increasingly displaced to the right of the value of log[A]_1_ = log[A]_2_ equal to the multiple of *RT*ln(10) for the respective $\Delta G_{\mathrm{Binding}}^{o}$.

The data plotted in figures 6 and 7*a,c,e* show the results obtained from three different virtual isoforms (three columns), differing from each other by their respective binding affinities. Nonetheless, the bottom panel of each column shows data pertaining to a single virtual isoform, demonstrating that the *cis-*force–flux relation for a particular enzyme is extremely variable, depending on the *trans-*substrate concentration. This, of course, is a trivial observation, but it raises doubt about the interpretation to be placed upon the shape of a force–flux relationship and its continuously variable ‘phenomenological coefficient’ expressing the continuously changing ratio of the flux to its respective force. By contrast, consider the interpretation to be placed on the eight different equilibrium slopes of the lines shown in figures 6*e,f* and 7*e*, and the fact that these slopes are apparently maximal when the binding affinity of the enzyme is closely ‘tuned’ to its equilibrium substrate concentration (cf. figures 2 and 3). Moreover, given that linear force–flux relations do not occur for this simplest of models of facilitated diffusion, it does not seem that linear force–flux relations are likely to be generally encountered elsewhere in experimental studies of biotransport mechanisms.^1^ Indeed, the application of LNET to chemistry, biochemistry and molecular physiology should never have been admissible. While linear force–flux relations have clear application to the work of electricians and plumbers, they have no place in any domain governed by the law of mass action. But, of course, it is far worse than that; the saturability of enzyme-catalysed reactions is yet another source of nonlinearity in biological force–flux relations, compounding the already inescapable nonlinear consequences of the law of mass action. Proponents of LNET might claim that the present results vindicate their expected near-equilibrium linearity for enzymes that are teleologically tuned to their respective substrate concentrations; but readers of LNET-inspired speculations are not generally informed about any kind of clearly defined or openly admitted domain of near-equilibrium validity.

Two features of some of the net rate curves shown in figures 6*e,f* and 7*e,f* warrant some cautionary remarks, particularly in relation to the uppermost clusters of curves arising from close to zero net rate. If such curves were actual experimental data, they might be taken as evidence of asymmetric diffusion on the one hand, or enzyme activation on the other.

Asymmetric diffusion is a difficult concept that has achieved some traction in the ‘origin-of-life literature’ [17] but is of no relevance to the present studies which are predicated on the applicability of the probability isotherm to any overall transport reaction and to its individual steps. In all the studies reported here, the overall equilibrium constant is unity for every condition simulated, meaning that the overall ratio of forward to backward transport rates is always equal to the *cis *: *trans* concentration ratio.

This leads immediately to the apparent depiction of enzyme activation in these same curves as they seem to rise *quasi*-exponentially from near-zero to reach their maximum slopes at their points of inflection. The portions of such curves lying to the left of their inflection points are similar in shape to those reported elsewhere from studies of the ΔpH-dependence of the rate of ATP synthesis by the F_o_F_1_-ATP synthase [18–20]. The authors of these studies were influenced by the LNET school, leading them to require an explanation for the fact that the curves were nonlinear; the explanation offered was that the pH gradient itself might serve to activate the enzyme. In the context of the present simulations depicted in figures 6 and 7, there is no enzyme activation because all eight rate coefficients are constant *within* any given curve, and they are also constant *between* the eight curves present in any given panel of graphs. The various shapes of all the curves shown in figures 6 and 7 are due solely to the playing out of the law of mass action as determining the unidirectional rates of each step and of the overall reaction in a saturable system. Upward concavity of force–flux relations in saturable systems, therefore, cannot be taken as evidence for enzyme activation in the absence of other kinetic and thermodynamic information.

### 5.6. Saturation kinetics and the Michaelis–Menten *K*_m_

Michaelis–Menten kinetic modelling has long afforded a useful basis on which to develop some simple principles of catalysis and to allow analytical solutions of various enzymatic scenarios to be obtained. The main problem with such models is that they are unable to afford any useful reconciliation between kinetics and thermodynamics, except at equilibrium. Indeed, the only so-called ‘thermodynamic’ equation that has appeared in these contexts with any regularity has been the van't Hoff isotherm, an expression more appropriately described as a ‘thermostatic’ equation. However, its thermodynamic equivalent form, the probability isotherm [8,10], leads directly to the powerful thermodynamically constrained kinetic equation, appropriately called the rate isotherm [8] and which is immediately available for use in thermodynamically constrained computer simulations of enzyme kinetics such as that developed here.

Figure 7 shows logarithmic (figure 7*a,c,e*) and linear (figure 7*b,d,f*) plots of the dependence on *cis*-substrate concentration, [A]_1_, of enzyme saturation (figure 7*a*,*b*), unidirectional forward rate (A_1→2_, figure 7*c*,*d*) and net reaction rate (A_1→2_–A_2→1_, figure 7*e*,*f*) for eight different values of *trans-*substrate concentration, [A]_2_, with $\Delta G_{\mathrm{Binding}}^{o}$ equal to –2 *RT*ln(10). In these graphs, the only curves that can be related to MMK are those for the unidirectional rate shown in figure 7*c*,*d*. In these instances, the logarithmic plots (figure 7*c*) show that the ‘*K*_m_ value’ for half-maximal unidirectional rate is relatively constant at approximately 10 mM [A]_1_ for all fixed values of [A]_2_. However, this does not correspond to any kind of association with 50% saturation of the enzyme except for the case of [A]_2_ = 10 mM, as is evident in the logarithmic saturation plots. Moreover, the concept of half-maximal rate finds resonance in only the *net* rate curves for the cases of −6 ≤ log[A]_2_ (M) ≤ −3.

While the data plotted in figure 7*c* suggest at least a near concordance with MMK for the *cis*-substrate concentration at which half-maximal unidirectional rates occur, this apparent ‘*K*_m_’ also varies somewhat with the fixed *trans*-substrate concentration at which the value is determined, and these situations do not correspond to 50% enzyme saturation. This is demonstrated by the data shown in table 5 for the values of [A]_1_ at which the half-maximal unidirectional rates occur. These values were determined with $\Delta G_{\mathrm{Binding}}^{o}$ set equal to −2*RT*ln(10) for two different values of *k*_4_ = *k*_−4_. The ‘*K*_m_’ was determined by forcing *R*_max_ on the enzyme by setting [*A*]_1_ = 10^5^ M and then finding the respective value of [A]_1_ to yield 0.5 *R*_max_. Thus, the apparent *cis*-‘*K*_m_’ varies with both *trans-*substrate concentration and with the relative rate-limitation among the steps.

**Table 5.** Estimate of virtual ‘*K*_m_’ for *cis*-substrate concentration [A]_1_ as influenced by rate limitation of reaction step 4 and *trans*-substrate concentration [A]_2_.

*k*_4_ = *k*_−4_ (s^−1^)	${\text{R}}_{\max}^{+}(\text{pmol}\;\text{c}{\text{m}}^{-2}\;{\text{s}}^{-1})$	[A]_2_ (mM)	[A]_1_ ‘*K*_m_’ (mM)	log[A]_1_ ‘*K*_m_’ (M)	saturation (%)
10^4^	0.4505	100	10.9910	−1.9590	72.3379
	2.3810	10	10.9524	−1.9605	51.1388
	4.1667	1	10.9167	−1.9590	31.4568
10^3^	0.0473	100	5.4167	−2.2663	52.8773
	0.3205	10	4.3590	−2.3606	38.3861
	0.7576	1	2.6667	−2.5740	13.8258

This kinetic behaviour generally lacks any useful correspondence with classical MMK. Although the saturation behaviour is roughly comparable, it finds no resonance in the corresponding rate behaviour as shown in figure 7. This suggests that Michaelis–Menten analysis is of limited value for understanding the physiological function of transporter enzymes either *in situ* or in the increasingly sophisticated nano-experimental protocols being used today. In particular, it seems unlikely that the classical Michaelis–Menten framework will be any more useful than the expectations of LNET in assisting with the design and interpretation of experiments in this expanding field.

### 5.7. Effect of *trans*-substrate concentration on *cis*-substrate transport

**Figure 8.**
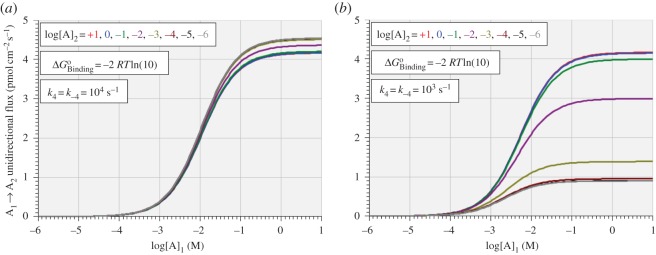
Variation of unidirectional flux rate A_1→2_ with *cis-*substrate concentration, [A]_1_, for eight different values of *trans-*substrate concentration, [A]_2_, with $\Delta G_{\mathrm{Binding}}^{o}$ fixed at –2 *RT*ln(10) and *k*_4_ = *k*_−4_ set to 10^4^ s^−1^ (*a*) and 10^3^ s^−1^ (*b*).

It is also shown in figure 7 that increasing the concentration of *trans*-substrate is inhibitory to both the unidirectional rate of *cis*-substrate transport and the net rate. However, this is not necessarily in conflict with the well-known observation that *trans*-substrate can be stimulatory towards unidirectional isotopic flux of *cis*-substrate, a phenomenon often referred to as *trans-*acceleration [13]. This is because the experimental measurement of unidirectional flux of *cis*-substrate is made, not across all of reaction steps 1–4, but only across steps 1–3. This distinction may be confirmed in the electronic supplementary material (the Excel file ‘INET.xlsx’) by contrasting the formula of cell F7 (overall unidirectional forward rate) with that of cell F8 (labelled ‘Isotopic A Influx across Steps 1 to 3’). Figure 8 shows the virtual experimental unidirectional flux rate, A_1→2_, as a logarithmic function of *cis-*substrate concentration, [A]_1_, for different values of *trans-*substrate concentration, [A]_2_, with $\Delta G_{\mathrm{Binding}}^{o}$ equal to –2 *RT*ln(10) and *k*_4_ = *k*_−4_ set to 10^4^ s^−1^ (figure 8*a*) and 10^3^ s^−1^ (figure 8*b*). These graphs show the effects of two different degrees of rate limitation of the conformational change in step 4 relative to that in step 2. In figure 8*a*, where *k*_4_ = *k*_−4_ is set to 10^4^ s^−1^, increasing the *trans*-substrate concentration has a small *negative* effect on unidirectional flux, while it has a marked *positive* effect if *k*_4_ = *k*_−4_ is set to 10^3^ s^−1^ (figure 8*b*). Thus, when there is no relative rate limitation of step 4, increasing the *trans*-substrate concentration is slightly inhibitory to the flux of *cis-*substrate, as shown by the small reductions in *R*_max_ as the *trans*-substrate concentration is raised from 1 µM to 10 M (figure 8*a*). On the other hand, when the rate-limitation of step 4 is present, this flux is greatly stimulated as *trans-*substrate is raised over the same domain (figure 8*b*). Note that the curves for the three highest *trans*-substrate concentrations form the lower (almost superimposed) cluster in figure 8*a*, while they form the upper cluster in figure 8*b*.

These results confirm that, in principle, *trans*-substrate can be either inhibitory or stimulatory on *cis*-substrate flux, depending on the presence or absence of rate limitation in the translocation of free enzyme relative to translocation of bound enzyme. When there is no relative rate limitation on the translocation of free enzyme, step 4, then *trans*-substrate competes for free enzyme on an equal footing with *cis*-substrate through the rapidly reversible step 3; under these conditions, *trans*-substrate can reduce the unidirectional flux of *cis*-substrate by reducing the availability of unbound E_2_ to participate in step 4 as the *trans*-substrate concentration increases. However, when step 4 is rate limiting, the quickest way to regenerate E_1_ from E_2_ to bind *cis*-substrate in step 1 is via reverse unidirectional fluxes of steps 3, 2 and 1 rather than from forward unidirectional flux of step 4; under these conditions, *trans*-substrate will increase the unidirectional flux of *cis*-substrate by increasing the availability of unbound E_1_ to participate in forward unidirectional flux through step 1.

It is worth noting that the *trans*-acceleration demonstrated for the present model arises purely from the relative rate-limitation of step 4 relative to step 2. Thus, while elsewhere it has been suggested that tetramer formation by the glucose transporter GLUT1 might be ‘important for understanding the *trans* acceleration observed for GLUT1-mediated transport’ [13], the present studies show that this is not a necessary condition for *trans*-acceleration.

### 5.8. Dissipation of free energy determined by the rate isotherm

The rate isotherm determines the kinetics of the actual reaction rates according to the second law and may be applied as such not only to the overall uniport process comprising steps 1–4, but also to the individual steps themselves. Thus, the free energy dissipation of the *i*th individual step, $\Delta G_{\mathrm{diss}}^{i}$, may be determined as

5.1 ΔGdissi=RTln ⁡rfirbi,

where $r_{f}^{i}$ and $r_{b}^{i}$ are the respective forward and backward reaction rates of the *i*th step. For the facilitated diffusion represented by steps 1–4, we have, at all times

5.2 ΔGdiss1=RTln ⁡k1[E1][A]1k−1[E1A],

5.3 ΔGdiss2=RTln ⁡k2[E1A]k−2[E2A],

5.4 ΔGdiss3=RTln ⁡k3[E2A]k−3[E2][A]2,

5.5 ΔGdiss4=RTln ⁡k4[E2]k−4[E1]

5.6 $$\begin{array}{rl} \text{and}\;\; & \Delta G_{\mathrm{diss}}^{1}+\Delta G_{\mathrm{diss}}^{2}+\Delta G_{\mathrm{diss}}^{3}+\Delta G_{\mathrm{diss}}^{4}=\Delta G_{\mathrm{diss}}^{A_{1\to 2}}=RT\ln\frac{{\left[\text{A}\right]}_{1}}{{\left[\text{A}\right]}_{2}}. \end{array}$$

**Figure 9.**
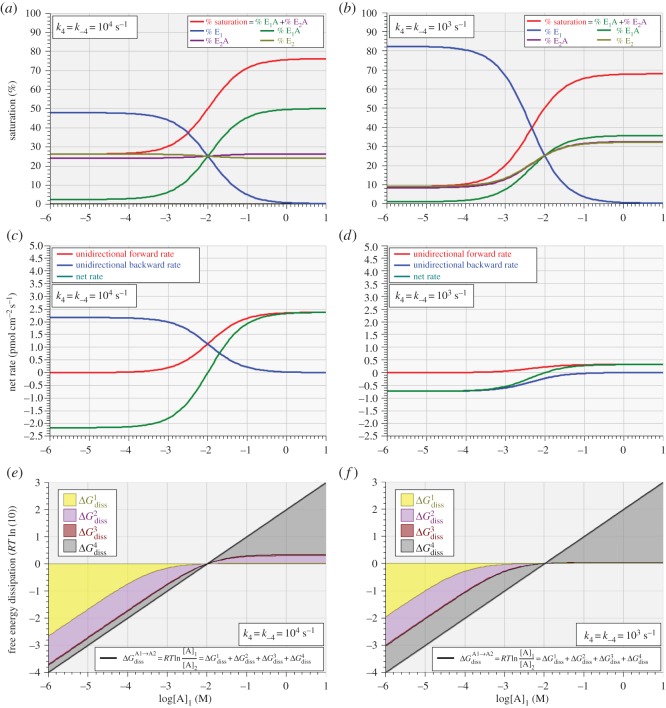
Variation of enzyme saturation (*a,b*), unidirectional and net reaction rates (*c,d*) and free energy dissipation (*e,f*) with *cis-*substrate concentration, [A]_1_, for *trans-*substrate concentration, [A]_2_ = 10 mM, and *k*_4_ = *k*_−4_ set to 10^4^ s^−1^ (*a,c,e*) and 10^3^ s^−1^ (*b,d,f*) and $\Delta G_{\mathrm{Binding}}^{o}=-2\;RT\text{ln(10)}$.

**Figure 10.**
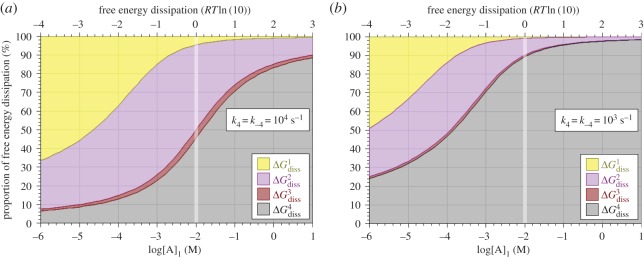
Variation of free energy dissipation for each of steps 1–4 as a proportion (percentage on the ordinate) of total free energy dissipation (top abscissa) with *cis-*substrate concentration, [A]_1_, for *trans-*substrate concentration, [A]_2_ = 10 mM, with *k*_4_ = *k*_−4_ = 10^4^ s^−1^ (*a*) and 10^3^ s^−1^ (*b*), and $\Delta G_{\mathrm{Binding}}^{o}=-2\;RT\text{ln(10)}$. The faded vertical panel at [A]_1_ = 10 mM indicates the indeterminate values of these proportions at thermodynamic equilibrium where total free energy dissipation is zero for [A]_1_ = [A]_2_ = 10 mM.

Figure 9 shows the results of simulations in which $\Delta G_{\mathrm{Binding}}^{o}$ was set at –2 *RT*ln(10) and the *trans-*substrate concentration, [A]_2_, fixed at 10 mM while the *cis*-substrate concentration, [A]_1_, was swept over the domain from 1 µM to 10 M. Figure 9*a,c,e* and *b,d,f* differs in that the rate coefficients for step 4 were set at 10^4^ s^−1^ (figure 9*a,c,e*) and 10^3^ s^−1^ (figure 9*b,d,f*). Figure 9*a,b* shows the variation in proportions of the four forms of the enzyme, including the total saturation, while figure 9*c,d* shows the overall unidirectional and net rates of transport. Figure 9*e,f* shows solutions to equation (5.6) as straight 45° lines indicating the total free energy dissipation, while solutions to equations (5.2)–(5.5) are shown as differently shaded areas indicating their respective contributions to the total free energy dissipation. Note that, in this context, negative free energy dissipation for overall transport from *cis*-compartment 1 to *trans-*compartment 2 simply means that the net transport is proceeding exergonically in the *reverse* direction whenever [A]_1_ < [A]_2_ as occurs for all values of [A]_1_ < 10 mM for the conditions represented in figure 9.

The dissipation plots shown in figure 9*e,f* demonstrate that there is no such thing as a unique rate-limiting step, even in this irreducibly simplified model of uniport. All that can be claimed with any consistency is that, for the conditions simulated, the dissociation of E_2_A in step 3 is the least rate-limiting of the four steps (indicated by very little free energy dissipation, $\Delta G_{\mathrm{diss}}^{3}$), while the translocation of bound enzyme in step 2 is significantly dissipative in both directions, and the translocation of free enzyme in step 4 becomes more dissipative as it is made more rate-limiting (figure 9*e,f*). Although the rate-limitation of step 4 forced in figure 9*b,d,f* resulted in much reduced reaction rates (figure 9*c,d*), the overall *molar rate* of free energy dissipation (figure 9*e,f*, 45° lines) is unaltered.

Nonetheless, the concept of ‘rate-limiting’ steps can be usefully quantified if the four reaction steps are regarded as chemical impedances in series, across which the chemical potential drops in steps, by analogy with the stepwise drop in electrical potential that occurs when electric current flows through a set of resistors in series. This idea is given graphical representation in figure 10, where the absolute free energy dissipations shown in figure 9*e,f* are re-drawn as *proportions* (i.e. percentages) of the total free energy dissipation determined by the *cis *: *trans* concentration ratio for substrate A. Figure 10*a* corresponds to figure 9*e*, i.e. that for which *k*_4_ = *k*_−4_ = 10^4^ s^−1^, while figure 10*b* corresponds to figure 9*f*, i.e. that for which *k*_4_ = *k*_−4_ = 10^3^ s^−1^.

As free energy dissipation at each step is an indicator of that step's chemical impedance, the proportional plots of figure 10 also provide a useful indication of the relative presence of rate-limiting behaviour for each of the four steps. As is to be expected from the rate coefficients shown in table 1, the translocation steps 2 and 3 are the most rate-limiting (highest impedance), except for step 1 at low *cis*-substrate concentrations. The main conclusion to draw from the free energy dissipation data shown in figures 9 and 10 is that the relative chemical impedance (rate-limitation) of any given step in an unbranched reaction sequence is a continuously variable function of substrate concentration, as are the unidirectional and net reaction rates.

Application of the probability isotherm affords a more realistic framework for developing the concept of rate-limiting steps for transport enzymes operating *in situ*. As demonstrated by the data shown in figure 10, the degree of rate limitation occurring in any given step can be quantified in terms of its instantaneous molar free energy dissipation as a proportion of that for the overall reaction.

### 5.9. Designing an optimal uniporter for a particular concentration gradient

The thinking behind the simulations in this section is unashamedly teleological, stimulated by the findings reported in the preceding sections. Taking overall unidirectional rate as a proxy for catalytic activity, it has been shown that the activity of a uniporter is highly sensitive to binding affinity, to translocational affinity and to substrate concentration, and that there is no simple correlation between the maximal unidirectional rate, *R*_max_, and enzyme saturation under a variety of scenarios. There is a functional advantage for cellular economy in optimizing *R*_max_ for a uniporter through the ‘design’ of its standard free energies of binding and translocation rather than through the membrane density of the enzyme. The data shown in figures 2 and 3 indicate that different concentrations of substrate require different thermodynamic and kinetic parameters for the enzyme to be optimally ‘tuned’ to any given situation, while the data shown in figure 5 indicate that any significant translocational affinity would be kinetically disadvantageous. Because any significant asymmetry of *cis*- and *trans-*binding affinity would require a compensatory asymmetry in the translocational affinity, resulting in kinetic disadvantage, we shall proceed on the assumption of no asymmetry in binding affinity and simply search for the optimal symmetric binding affinity for transport down four different concentration gradients.

**Figure 11.**
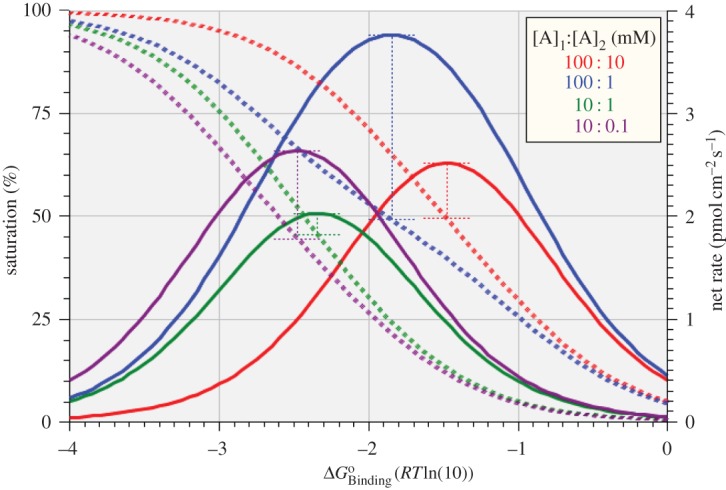
Variation of enzyme saturation (left axis, dashed lines) and net rate of transport down four different concentration gradients for substrate (right axis, solid lines) with $\Delta G_{\mathrm{Binding}}^{o}$. The positions of *R*_max_ and the corresponding binding affinities and saturation levels are marked as recorded in table 6.

The results of these simulations are shown in figure 11 for enzyme saturation (left axis and dashed lines) and net transport rate (right axis and solid lines) plotted against the standard free energy of binding. The results are shown in four different colours, corresponding to four different *cis*-[A]:*trans*-[A] concentration gradients as given in the legend. The coordinate points for optimal rate (*R*_max_) and the levels of binding affinity and enzyme saturation at which they are achieved, are all marked on figure 11 and recorded in table 6. These data demonstrate that, as for rapid equilibration between compartments, transport down a concentration gradient requires fine tuning of the binding affinity for optimum performance. Unsurprisingly, the optimal binding affinity for a particular concentration gradient is found to lie between the optimal affinities for rapid equilibration of the respective concentrations (figure 2 and table 3), and is generally less than the mean of the two affinities.

**Table 6.** Optimal standard free energy of binding ($\Delta G_{\mathrm{Binding}}^{o}$), optimal net rate of substrate transport (*R*_max_) and respective enzyme saturation obtained for four different *cis*-[A] : *trans*-[A] gradients.

*cis-*[A] : *trans-*[A] (mM)	log *cis-*[A] : *trans-*[A] (M)	$\Delta G_{\mathrm{Binding}}^{o}(RT\text{ln( 10) })$	*R*_max_ (pmol cm^−2^ s^−1^)	saturation (%)
100 : 10	–1 : –2	–1.4773	2.5191	49.4385
100 : 1	–1 : –3	–1.8484	3.7684	49.2387
10 : 1	–2 : –3	–2.3389	2.0302	45.4889
10 : 0.1	–2 : –4	–2.4773	2.6381	44.6689

### 5.10. Factors contributing to the kinetic performance of a uniporter

The simulations described above demonstrate that optimal ‘tuning’ of a simple four-step uniporter enzyme for a particular task, e.g. uniport down a relatively constant concentration gradient, involves the possible adjustment of a complex set of thermodynamic and kinetic parameters, thus

(a) Thermodynamic parameters:
  — Affinity of *cis*-binding at step 1
  — Intrinsic probability ratio for the two bound states of the enzyme at step 2
  — Affinity of *trans*-binding at step 3
  — Intrinsic probability ratio for the two free states of the enzyme at step 4
  — Binding imbalance between steps 1 and 3
  — Translocation imbalance between steps 2 and 4
(b) Kinetic parameters:
  — Assumed upper limit for the second-order diffusion-limited association coefficients (*k*_1_ and *k*_−3_)
  — Assumed upper limit for the first-order translocation constants (*k*_2_, *k*_−2_, *k*_4_ and *k*_−4_)
  — Relative rate limitation of steps 2 and 4, i.e. *k*_4_ relative to *k*_2_.

Added to these relatively simple considerations is the possibility, even the probability, that some of these parameters may be voltage-dependent as the three-dimensional structure of the protein adapts to physiological differences in transmembrane potential (e.g. allowing glucose influx to increase in response to depolarization arising from metabolic or circulatory insufficiency).

In considering the thermodynamics and kinetics of transporters operating *in situ*, it is important to account for anisotropy in every aspect, whether in theoretical concepts, experimental protocols or physiological boundary conditions involving transmembrane electrical and chemical gradients. Very few of these aspects are comprehended within the traditional approaches of LNET or MMK; however, they are all comprehended within the approach of INET, based as it is on the law of mass action constrained by the probability isotherm.

From one point of view, the virtual experimental results reported here pertain to idealized abstractions, with only the broadest attempt to relate the absolute values of any of the kinetic constants or substrate concentrations to actual experimentally determined values; only the thermodynamic constraints on such kinetic constants have been strictly honoured. Nonetheless, a consistent pattern of results has emerged, indicating that unidirectional and net rates of a simple uniport reaction are exquisitely sensitive to the above-listed thermodynamic and kinetic factors. Put another way, the thermodynamic and kinetic parameters of an enzyme may be expected to be ‘tuned’ to its specific physiological function *in situ*. Moreover, slow changes in membrane potential may influence the manifestation of both anisotropy in binding affinity and anisotropy in the shuttling of bound and unbound forms of the enzyme, leading to voltage-dependent inhibition or activation of transport. Different situations will call for different thermodynamic and kinetic ‘tuning’, such as might occur among the many isoforms that are known to exist for enzymes that transport the same molecule in different anatomical locations with different physiological boundary conditions. In this light, the abundance of information on the numerous isoforms of glucose transporters [13,21,22] would seem to provide much fertile ground for application of the INET methods demonstrated here, constrained by the probability isotherm.

## 6. Conclusion

The purpose of this study was to explore what kinds of insights might be gained by applying classical kinetic concepts, such as the law of mass action, to explicit molecular models of membrane transport constrained by the probability isotherm. This had not been possible in the world that existed before the advent of high-speed digital computers. In those days, it was necessary to resort to simplifying assumptions of one kind or another to deal with kinetic models for which there were no ready-made analytical solutions available from mathematics. Therefore, it was that investigators came to rely on the Michaelis–Menten approach to enzyme kinetics on the one hand [22], and on the near-equilibrium linear force–flux assumptions of LNET, on the other [16].

Against this pre-digital backdrop, the present paper provides an extremely belated unveiling of the possibilities that may arise if the design and interpretation of the increasingly ingenious nano-experiments being performed around the world were to be informed by what may reasonably be called the emergent discipline of INET. INET may be distinguished in the following ways:

— INET is intuitive because it is based on familiar classical concepts in chemical kinetics, such as the law of mass action;
— INET is non-equilibrium in two senses, because:
  it deals with unidirectional and net rates both at the level of the elementary step and at the level of the overall multi-step reaction, and
  its application is valid under all conditions, whether they be very far from equilibrium, close to equilibrium or even precisely at thermodynamic equilibrium;
— INET is thermodynamic in that it recognizes the second law constraint on all forms of kinetic modelling that inheres in the probability isotherm and its kinetic equivalent, the rate isotherm. This effectively neutralises the unhelpful myth that thermodynamics has nothing to say about kinetics except at equilibrium.

The kinetic model studied in this paper is irreducibly minimalist as a model of facilitated diffusion, yet it has already indicated essential complexities of real enzyme behaviour that are not adequately accounted for by MMK in the pedagogical literature [22] or LNET principles in the research literature [16]. And these complexities are formidable, even without bringing enzyme mobilization, multimeric cooperativity or gene expression to account [21,22]. However, the list of adjustable parameters summarized above is already sufficiently large to make the model of very limited specific predictive value on its own. All that the present study can indicate is the breadth of the range of integrated kinetic and thermodynamic considerations that can, and should, be brought to the design and interpretation of experiments in biotransport enzymology. Further development of such models should ideally proceed closely in tandem with experimental work, either re-interpreted retrospectively [10,11] or designed prospectively, and be informed by the principles of INET, constrained everywhere and always by the probability isotherm.

It is also possible that, informed by this INET approach, we might begin to gain insight into the factors that determine the molecular behaviour of transport enzymes operating across the very strong transmembrane electric fields that are generally present. What actually *constrains* uniporters to do what they do (binding reactions, conformational flips, etc.) while obeying the second law exactly so as to ensure compliance with the constraint that the overall Δ*G*^o^ remains zero over the entire cycle of reaction steps? The second law is an (apparently) empirical fact, deriving from no known ‘laws’ more fundamental than itself, but its iron rule constrains even the most Heath Robinsonian collections of transmembrane amino acid sequences as they undergo their conformational changes and reveal their respective *cis*- and *trans*-binding affinities in accordance with their physiological functions. The second law informs us that this must be so and, though we accept this by inductive faith, we do not yet apprehend the mechanism by which this is determined.

## Supplementary Material

Numerical simulation of 4-step facilitated diffusion model

## Footnotes

1 It is also straightforward to demonstrate the continuous variability of net rate (flux) with composition at constant thermodynamic force by sweeping the *cis-* and *trans*-substrate concentrations while holding their ratio constant, thereby demonstrating, once again, the misplaced expectation of LNET in a domain ruled by the law of mass action.

## Data accessibility

All data were created using the Excel file (INET.xlsx) supplied as the electronic supplementary material. The numerical solutions were not stored but rather plotted graphically and screen captured for reproduction as text figures. The text figures are supplied as separate image files.
